# X-linked inhibitor of apoptosis positive nuclear labeling: a new independent prognostic biomarker of breast invasive ductal carcinoma

**DOI:** 10.1186/1746-1596-6-49

**Published:** 2011-06-07

**Authors:** Yutao Zhang, Jianhua Zhu, Yun Tang, Feng Li, Hongyuan Zhou, Bofang Peng, Chifeng Zhou, Rong Fu

**Affiliations:** 1 Department of Pathology, The First People' s Hospital of Zigong, Zigong, China

**Keywords:** XIAP nuclear labeling, Smac, apoptosis index, prognosis

## Abstract

**Background:**

It's well recognized that X-linked inhibitor of apoptosis (XIAP) was the most potent caspase inhibitor and second mitochondria-derived activator of caspase (Smac) was the antagonist of XIAP. Experiments in vitro identified that down regulation of XIAP expression or applying Smac mimics could sensitize breast cancer cells to chemotherapeutics and promote apoptosis. However, expression status and biologic or prognostic significance of XIAP/Smac in breast invasive ductal carcinoma (IDC) were not clear. The present study aimed to investigate relationship among expression status of XIAP/Smac, apoptosis index (AI), clinicopathologic parameters and prognosis in IDC.

**Methods:**

Immunohistochemistry and TUNEL experiment were performed to detect expression of XIAP, Smac, ER, PR, HER2 and AI in 102 cases of paraffin-embedded IDC samples respectively. Expression of XIAP/Smac were also detected in limited 8 cases of fresh IDC specimens with Western blot.

**Results:**

Positive ratio and immunoscore of XIAP was markedly higher than Smac in IDC (*P *< 0.0001). It was noteworthy that 44 cases of IDC were positive in nuclear for XIAP, but none was for Smac. Expression status of Smac was more prevalent in HER2 positive group than negative group (*P *< 0.0001) and AI was positively correlated with HER2 protein expression (r_s _= 0.265, *P *= 0.017). The present study first revealed that XIAP positive nuclear labeling (XIAP-N), but not cytoplasmic staining (XIAP-C), was the apoptotic marker correlated significantly with patients' shortened overall survival (*P *= 0.039). Survival analysis demonstrated that XIAP-N was a new independent prognostic factor except for patient age and lymph node status.

**Conclusion:**

Disturbed balance of expression between XIAP and Smac probably contributed to carcinogenesis and XIAP positive nuclear labeling was a new independent prognostic biomarker of breast IDC.

## Background

Disequilibration between cell proliferation and apoptosis has been identified for a momentous mechanism of tumorigenesis. Balance between expression status of anti-apoptotic and pro-apoptotic proteins determines cells to be alive or not. The key event of apoptosis occurrence is cascade activation of caspases, and inhibitor of apoptosis proteins (IAPs) play a important role in caspase inhibition. It is well recognized that XIAP is the most potent caspase inhibitor and Smac is one of the antagonists of XIAP. Unbalanced expression between XIAP and Smac probably contributes to progression of renal cell carcinomas and results in marked apoptosis resistance of this tumour[1]. Breast cancer is the most common malignant tumour of female and estimated new cases in America are 192,370 in 2010[2]. Previous experiments in vitro have identified that sustained overexpression of XIAP can cause acquired tumor necrosis factor-alpha related apoptosis-inducing ligand (TRAIL) resistance in MDA-231 human breast cancer cell[3]. Down regulation of XIAP expression or applying exogenous Smac mimics can sensitize tumor cells, especially for breast cancer cells, to chemotherapeutics and promote apoptosis[4-12]. IDC, not otherwise specified, is the most frequent histological subtype of breast cancer. However, expression status and biologic or prognostic significance of XIAP/Smac proteins in breast IDC are not clear. Immunohistochemistry and western blot are performed to detect expression of XIAP/Smac and terminal TdT-mediated dUTP nick-end labeling (TUNEL) method is performed to detect AI in IDC in the present study. And then, relationship among expression status of those proteins, AI, clinicopathologic parameters and prognosis is analyzed.

## Materials and methods

### Patients and Tissue samples

This study was done with IRB approval and all patients' consent. Formalin-fixed, paraffin-embedded 102 cases of consecutive IDC samples with different grades and stages (Table 1) were obtained from patients who had received modified radical mastectomy in the authors' institution. The haematoxylin-eosin staining sections had been checked by two experienced pathologists before experiment. All of the patients were not administered any treatment before operation and received postoperative chemotherapeutics (Paclitaxel + Adriamycin + Cyclophosphamide) for 15 consecutive weeks. And 9 out of the 102 patients still received radiotherapy in addition. Limited 8 cases of fresh IDC specimens were obtained from Laboratory of Pathology of West China Hospital.

**Table 1:** Pathological staging and grading of 102 cases of invasive ductal carcinoma

staging		grading	
pT1	19(18.6%)	G1	25(24.5%)
pT2	57(55.9%)	G2	50(49.0%)
pT3	24(23.5%)	G3	27(26.5%)
pT4	2(2.0%)		
Total	102(100%)		102(100%)

### Antibodies

The following antibodies at indicated dilutions were used in our study: XIAP (rabbit polyclonal, ABZOOM, USA, 1:100 for IHC, and 1:1000 for immunoblotting), Smac (mouse monoclonal, Cell Signaling, USA, 1:100 for IHC, and 1:1000 for immunoblotting), ER and PR (rabbit monoclonal, MAIXIN, Fujian, China), HER2 (mouse monoclonal, MAIXIN, Fujian, China), GAPDH (mouse monoclonal, clone 6C5, Kangcheng, Shanghai, China, 1:10000 for immunoblotting).

### Immunohistochemistry

Sections (4 μm) were immunostained by standard SP method protocol. H_2_O_2 _(0.3%) was employed to block endogenous peroxydase-binding activity. Antigen retrieval was by microwave boiling in citrate buffer (pH 6.0) for 12 min. Omission of primary antibodies was used as a blank control. Human normal skeletal muscle and adenocarcinoma of stomach tissue sections were immunostained as positive control for XIAP and Smac antibodies respectively. Immunostaining was evaluated and scored by two experienced pathologists independently. All the staining was scored in epithelial cells, but not in stromal cells or inflammatory cells. Cytoplasmic staining of XIAP/Smac and HER2 showed a diffuse staining pattern when positive and was scored by conventional four-tiered semiquantitative scoring system (scores 0-3 for negative, weak, moderate, and strong staining, respectively) based on staining intensity[13]. XIAP was detected in nucleus and cytoplasm, and these results were scored as XIAP-N and XIAP-C separately. The staining of hormone receptor markers ER and PR was exclusively in nucleus. It was assessed as positive that more than 10% tumor cells showed brown nucleus during ER, PR and XIAP-N immunostaining.

### Western Blot Analysis

Fresh frozen tissue samples were minced and grinded down to powder with mortar and pestle on liquid nitrogen. Total proteins from powdered tissue samples were extracted in the presence of protease inhibitor cocktails (Roche Diagnostics, Mannheim, Germany), quantitated by using the BCA kit (Pierce Biotechnology Inc., Rockford, IL, USA) and resolved by 10% SDS polyacrylamide (Sigma, St Louis, MO, USA) gel electrophoresis. Proteins were electroblotted to PVDF membrane (Amersham Biosciences UK Ltd., Little Chalfont, UK) in CAPS buffer (pH 11.0) (Amresco, Solon, OH, USA), and then incubated with block solution (5% non-fat milk, 0.1% Tween 20, in 1×TBS, Sigma, St Louis, MO, USA) at room temperature for 2 h. Anti-GAPDH was used as internal control. Horseradish peroxidase-labeled secondary antibodies were from Zymed (San Francisco, CA, USA). Incubation with primary and secondary antibodies were at room temperature for 2 h and 1.5 h, respectively. Signals were detected by exposure to X-ray films after treatment with the Super Signal enhanced chemiluminescence kit (Pierce Biotechnology Inc., Rockford, IL, USA) after incubation with primary and secondary antibodies.

### TUNEL assay

Apoptotic tumor cells were detected with TUNEL method, using an in situ cell death detection kit (Roche Diagnostics, Mannheim, Germany). The assay was performed according to the manufacturer's instructions. Briefly, after routine deparaffinization and treatment with H_2_O_2 _(3%), sections were digested with proteinase K (20 μg/ml, pH 7.4, 12 min) at 25°C and incubated with the reaction mixture (1:40, 60 min) at 37°C. Incorporated fluorescein was detected with horseradish peroxidase after a 30 min incubation at 37°C and subsequent dyed with DAB. Brown nucleus was assessed as positive apoptotic cell and counted for 1000 tumor cells, scoring as AI in one section for at least 10 high power fields.

### Statistical Analysis

General statistical and survival analysis were carried out with the statistical software package SPSS 17.0 (SPSS, Chicago, USA). Intergroup differences were examined by using *x*^2 ^test, independent example *t*-test and Mann-Whitney *U*-test. A *P *-value of less than 0.05 was considered to indicate the statistical significance.

## Results

### Relationship among expression status of XIAP/Smac detected by immunohistochemistry, clinicopathologic parameters and biomarkers

**Figure 1:**
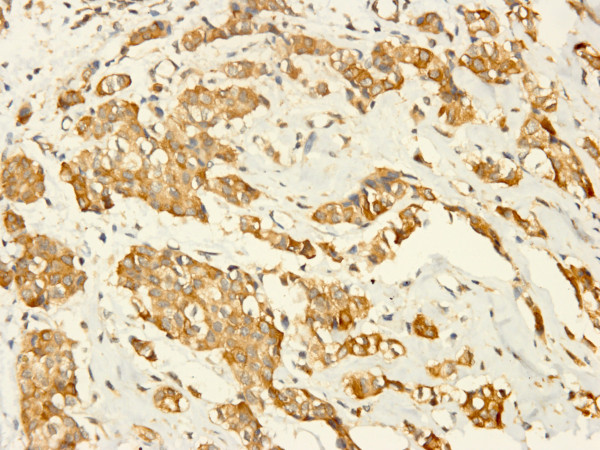
**Higher power view of representative cytoplasmic immunostaining of XIAP in IDC**. Original magnification for this figure: ×400.

**Figure 2:**
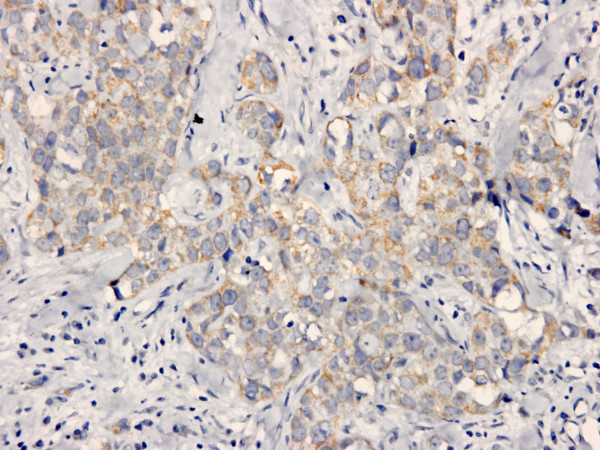
**Higher power view of representative cytoplasmic immunostaining of Smac in IDC**. Original magnification for this figure: ×400.

**Figure 3:**
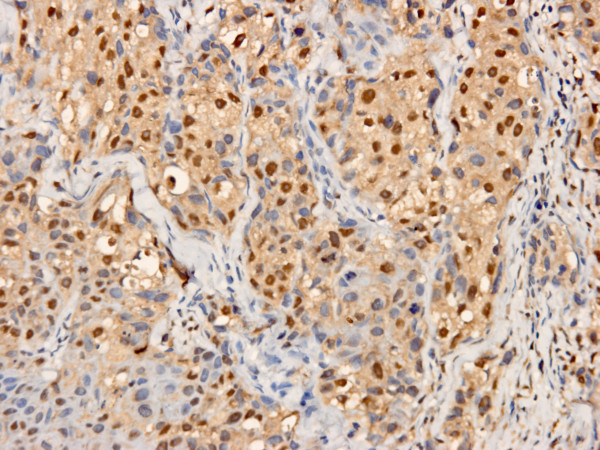
**Higher power view of representative nuclear immunostaining of XIAP, in addition to cytoplasmic staining in IDC**. Original magnification for this figure: ×400.

Both XIAP and Smac were positive in cytoplasm of tumor cells with strong or moderate intensity, respectively (Figure 1, 2). The positive ratio of XIAP (84.3%, 86/102) was more higher than that of Smac (33.3%, 34/102), and immunoscore of XIAP was higher than Smac in IDC too (*P *< 0.0001). It was noteworthy that 44 IDC samples were nuclear positive for XIAP (Figure 3), but none was for Smac. And cytoplasm positive status of XIAP nuclear positive group was stronger than the negative group (*P *= 0.030, 0.047) (Table 2, 3). Otherwise, Smac immunoscore was prevalent in HER2 positive group than negative group (*P *< 0.0001). Remaining data revealed that the expression status of XIAP/Smac was not correlated with patient age, tumor size, lymph node status, histologic grading, expression of ER and PR (Table 2, 3).

**Table 2:** Relationship analysis between positive rate of XIAP/Smac and clinicopathologic variables

	n*	XIAP-C		Smac		XIAP-N	
				
		PR	*P *value	PR	*P *value	PR	*P *value
**Age**							
<50	49	42(85.7%)		20(40.8%)		21(42.9%)	
≥50	53		0.789		0.145		1.000
**Size**		44(83.0%)		14(26.4%)		23(43.4%)	
≤5 cm	77	67(87.0%)		26(33.8%)		33(42.9%)	
>5 cm	25	19(76.0%)	0.212	8(32.0%)	1.000	11(44.0%)	1.000
**LN status**							
N_0_	39	33(84.6%)		14(35.9%)		14(35.9%)	
N_1-3_	63	53(84.1%)	1.000	20(31.7%)	0.672	30(47.6%)	0.305
**Grade**							
I	25	22(88.0%)		10(40.0%)		13(52.0%)	
II-III	77	64(83.1%)	0.755	24(31.2%)	0.468	31(40.3%)	0.356
**ER**							
positive	39	31(79.5%)		14(35.9%)		20(51.3%)	
negative	63	55(87.3%)	0.401	20(31.7%)	0.672	24(38.1%)	0.221
**PR**							
positive	44	36(81.8%)		18(40.9%)		23(52.3%)	
negative	58	50(86.2%)	0.591	16(27.6%)	0.204	21(36.2%)	0.112
**HER2**							
positive	94	78(83.0%)		34(36.2%)		41(43.6%)	
negative	8	8(100.0%)	0.351	0(0.0%)	**0.049**	3(37.5%)	1.000
**XIAP-N**							
positive	44	41(93.2%)		16(36.4%)		**/**	
negative	58	44(75.9%)	**0.030**	18(31.0%)	0.672	**/**	

n*: number of cases. PR: positive rate. LN: lymph node. XIAP-C and XIAP-N denoted cytoplasmic immune staining score and nuclear labeling of XIAP, respectively. *P*-values (two sided) < 0.05 were highlighted in bold.

**Table 3:** Relationship analysis among XIAP/Smac immunoscore, apoptosis index, and clinicopathologic variables

	n*	XIAP	*P *value	Smac	*P *value	AI	*P *value
**Age**							
<50	49	2.3 ± 0.8		0.5 ± 0.6		1.3 ± 1.0	
≥50	53	2.4 ± 0.8	0.586	0.3 ± 0.5	0.124	1.3 ± 1.1	0.903
**Size**							
≤5 cm	77	2.4 ± 0.8		0.4 ± 0.6		1.3 ± 1.1	
>5 cm	25	2.2 ± 0.9	0.386	0.4 ± 0.7	0.880	1.4 ± 1.0	0.849
**LN status**							
N_0_	39	2.4 ± 0.8		0.4 ± 0.6		1.2 ± 0.8	
N_1-3_	63	2.4 ± 0.8	0.969	0.4 ± 0.6	0.958	1.4 ± 1.2	0.282
**Grade**							
I	25	2.5 ± 0.7		0.5 ± 0.6		1.6 ± 1.4	
II-III	77	2.3 ± 0.8	0.444	0.4 ± 0.6	0.555	1.2 ± 0.9	0.227
**ER**							
positive	39	2.3 ± 0.8		0.4 ± 0.6		1.2 ± 1.2	
negative	63	2.4 ± 0.8	0.376	0.4 ± 0.6	0.659	1.4 ± 1.0	0.463
**PR**							
positive	44	2.3 ± 0.8		0.5 ± 0.6		1.3 ± 0.8	
negative	58	2.4 ± 0.8	0.669	0.3 ± 0.6	0.319	1.4 ± 1.2	0.779
**HER2**							
positive	94	2.4 ± 0.8		0.4 ± 0.6		1.3 ± 0.9	
negative	8	2.2 ± 0.8	0.544	0.0 ± 0.6	**<0.0001**	1.8 ± 2.1	0.611
**XIAP-N**							
positive	44	2.6 ± 0.6		0.4 ± 0.6		1.3 ± 1.1	
negative	58	2.2 ± 0.9	**0.047**	0.3 ± 0.6	0.540	1.4 ± 1.0	0.630

n*: number of cases. AI: apoptosis index. LN: lymph node. XIAP-C and XIAP-N denoted cytoplasmic immune staining score and nuclear labeling of XIAP, respectively. *P*-values (two sided) < 0.05 were highlighted in bold.

### Western Blot detection of XIAP/Smac protein expression

**Figure 4:**
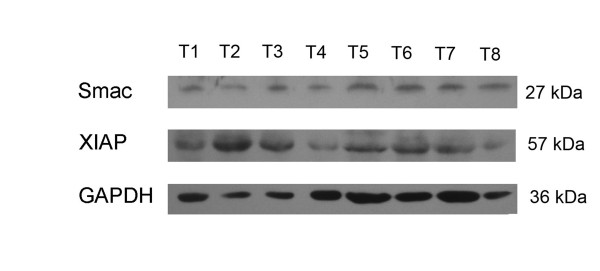
**XIAP and Smac proteins in IDC (T1-T8) assessed by Western blot analysis**. GAPDH served as internal control of protein loading. Expression of XIAP was more stronger than Smac in IDC with semi-quantitation analysis.

In limited 8 cases of fresh IDC examples, we detected expression of both XIAP/Smac protein and GAPDH internal control (Figure 4). The semi-quantitation analysis data indicated that expression status of XIAP protein was more stronger than Smac in fresh IDC specimens with ImageQuant software (Data wasn't shown.).

### Relationship among apoptosis index, clinicopathologic parameters and biomarkers

**Figure 5:**
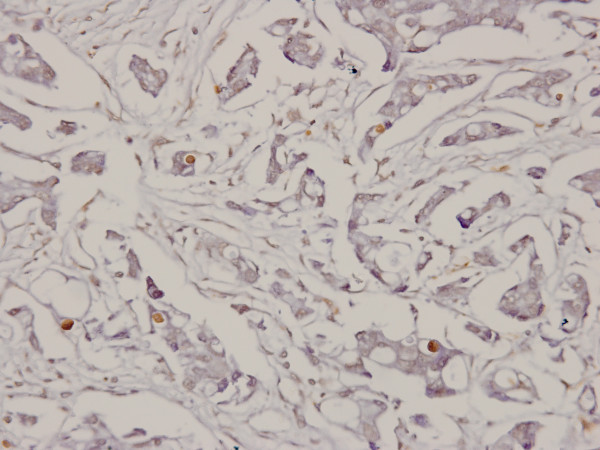
**Representative positive apoptotic tumor cells in IDC by TUNEL detection**. Original magnification for this figure: ×400.

The positive apoptotic tumor cells showed brown nucleus in TUNEL detection (Figure 5). AI of total 102 IDC examples variated from 0.212 to 6.044 (mean value 1.322 ± 1.052). The correlation analysis revealed that AI was positively correlated with HER2 protein expression (r_s _= 0.265, *P *= 0.017), but not correlated with patient age, tumor size, lymph node status, histologic grading and expression of XIAP, Smac, ER and PR (Data wasn't shown.).

### Relationship among expression status of XIAP/Smac, apoptosis index, clinicopathologic parameters and prognosis in IDC

**Figure 6:**
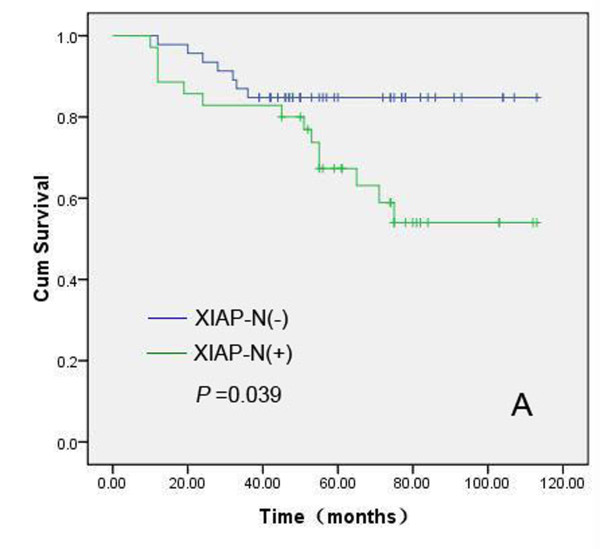
**Kaplan-Meier comparison result showed that XIAP-N was correlated significantly with IDC patients' shortened overall survival**. Log rank test *P*-value (two sided) was listed in the figure.

**Figure 7:**
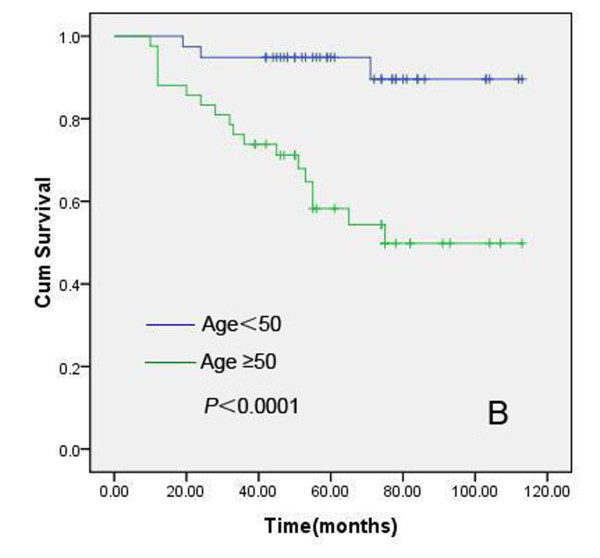
**Kaplan-Meier comparison result showed that patient age (≥50 years) was correlated significantly with IDC patients' shortened overall survival**. Log rank test *P*-value (two sided) was listed in the figure.

**Figure 8:**
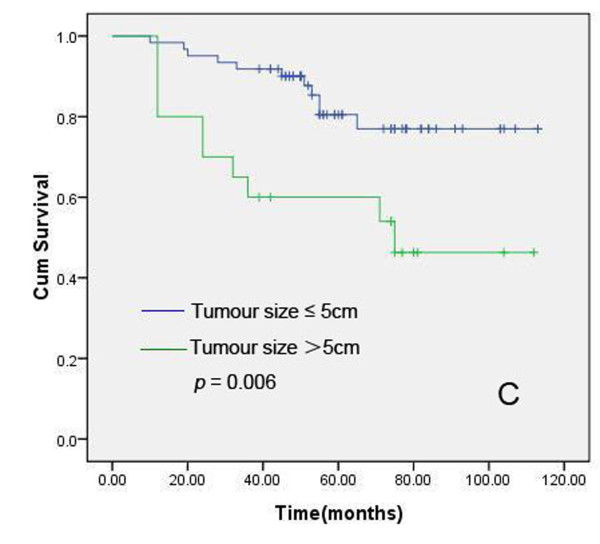
**Kaplan-Meier comparison result showed that tumor size (>5 cm) was correlated significantly with IDC patients' shortened overall survival**. Log rank test *P*-value (two sided) was listed in the figure.

**Figure 9:**
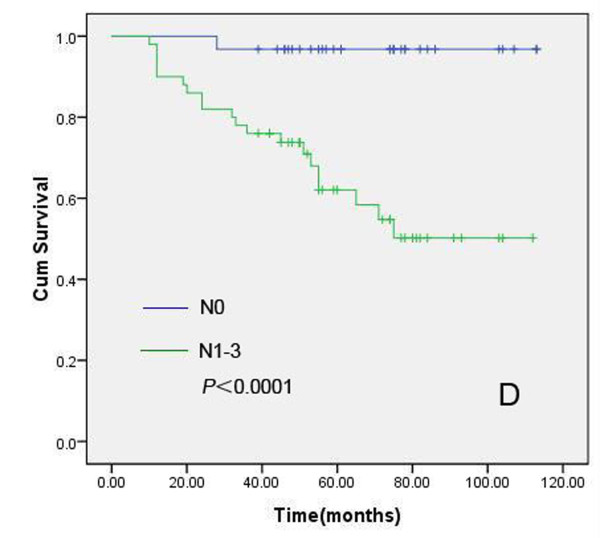
**Kaplan-Meier comparison result showed that lymph node status was correlated significantly with IDC patients' shortened overall survival**. Log rank test *P*-value (two sided) was listed in the figure.

We further analyzed the prognostic value of XIAP-N, XIAP-C, Smac, HER2, ER, PR, AI, patient age, lymph node status and histologic grading in this cohort of 102 pathologically confirmed breast IDC patients, who received modified radical mastectomy, postoperative chemotherapeutics or radiotherapy. The median follow-up time for all patients was 60.0 months. In this cohort, 27 patients died of recurrence or metastases, and another three patients who had been pathologically confirmed recurrence were alive. Kaplan-Meier method and log rank test were used for univariate analysis of overall survival and Cox proportional hazard regression was used for multivariate analysis. Univariate analyses revealed that XIAP-N (positive vs negative), patient age (<50 years vs ≥50 years), tumor size (<5 cm vs ≥5 cm) and lymph node status (N_0 _vs N_1-3_) had prognostic significance (Figure 6, 7, 8 and 9). However, in multivariate analysis incorporating these parameters, only XIAP-N, patient age and lymph node status retained independent prognostic power, with approximately 3.0-, 9.2- to 14.8-fold increase of risk for disease-specific death respectively (Table 4).

**Table 4:** Relationship analysis among XIAP/Smac expression, apoptosis index, clinicopathologic parameters and prognosis

	n*	*P value*	*P value*	*RR*	95%CI	
					
		(Log rank)	(Cox-reg)		Lower	Upper
**Age**						
<50	49					
≥50	53	**<0.0001**	**0.002**	9.181	2.332~36.147	
**Size**						
≤5 cm	77					
>5 cm	25	**0.006**	0.065	2.903	0.935~9.016	
**LN status**						
N_0_	39					
N_1-3_	63	**<0.0001**	**0.012**	14.757	1.803~120.750	
**Grade**						
I	25					
II-III	77	0.596	0.391	0.555	0.144~2.134	
**XIAP-C**						
<2	16					
≥2	86	0.153	0.188	0.398	0.101~1.568	
**XIAP-N**						
positive	44					
negative	58	**0.039**	**0.043**	3.027	1.033~8.869	
**Smac**						
<2	68					
≥2	34	0.595	0.395	0.634	0.222~1.809	
**ER**						
positive	39					
negative	63	0.965	0.774	1.181	0.379~3.681	
**PR**						
positive	44					
negative	58	0.861	0.779	1.177	0.378~3.662	
**HER2**						
<2	31					
≥2	71	0.182	0.082	3.522	0.853~14.538	
**AI**						
<1.32	65					
≥1.32	37	0.648	0.658	1.244	0.474~3.264	

n*: number of cases. RR: relative risk. CI: confidence interval. LN: lymph node. AI: apoptosis index. XIAP-C and XIAP-N denoted cytoplasmic immune staining score and nuclear labeling of XIAP, respectively. *P*-values (two sided) < 0.05 were highlighted in bold.

## Discussion

XIAP was the most potent caspase inhibitor, whose molecular structure was known best in IAP family[14]. There were three important components in XIAP gene structure, including BIR domain, Linker domain and Ring-figure domain which possesed the activity as ubiquitin ligase E3, and XIAP could bind to caspase-3, 7, 9 directly to inhibit their activity[15]. Expression of XIAP protein had been detected in most of carcinoma cells, and overexpression of this protein was correlated with patients'sensitivity to anticancer drugs and prognosis[16]. For instance, overexpression of XIAP and Survivin, another member of IAP family, resulted in severe resistance to Adriamycin, Paclitaxel and Vincristine in several kinds of breast cancer cell[17,18]. In LNCaP prostate cancer cells, XIAP restrained apoptosis induced by Paclitaxel through cutting down activity of caspase-3 and inhibiting processing of pro-caspase-3 moreover[19]. On the contrary, patients of acute myeloid leukaemia with low expression level of XIAP could get more favorite prognosis[16].

Smac, which was named by direct IAP-binding protein with low pI (DIABLO) too, was one of the two known negative regulators of XIAP presently. Smac promoted apoptosis through several ways, including firstly interacting with cytochrome c/Apaf-1/pro-caspase-9 complex to activate caspase-9 or downstream effector molecule caspase-3[20,21], secondly binding to BIR domain of XIAP competively and blocking it's inhibitory effect on caspases[22], and lastly cooperating with Omi/HtrA2 to promote XIAP to degradation[23,24]. Previous study reported that total positive ratio of Smac was 62% in several carcinomas, and lack of Smac would lead to down regulation of apoptosis[25].

Preliminary research showed positive ratio of XIAP in breast cancer were 89.7%, but no related report about Smac[26]. Our data indicated not only positive ratio but also immunoscore of XIAP were more higher than Smac in breast IDC, and semi-quantitation analysis of western blot detection proved it too. But it just could be regarded as a attempt because the fresh samples were so limited in this study. Disturbed balance of expression between XIAP and Smac contributed to progression of renal cell carcinoma and XIAP was an independent prognostic biomarker of clear cell renal cell carcinoma[1,27]. Similarly, it also could be believed that disturbed balance of expression between XIAP and Smac contributed to carcinogenesis of IDC based on results of the present study. A set of data, consisting with other report, showed that expression status of XIAP/Smac was not correlated with patient age, tumor size, lymph node status, histologic grading, expression of ER and PR[26]. Whereas, Jaffer and his colleagues found a possible role of XIAP in the more aggressive clinical behavior of grade 3, compared with lower-grade ductal carcinomas[28]. These conflicting results need be confirmed in a following large sample study.

In previous reports, XIAP was only detected in cytoplasm[26,28]. However, we found 44 IDC samples were positive in nucleus and cytoplasm for XIAP simultaneously, but none was for Smac. This difference was maybe caused by using different primary antibodies and tissue specimens from different race. Tissue specimens in present study were all from Chinese female patients, but not from Europe or America. Something different from previous reports was that XIAP nuclear labeling (XIAP-N), but not cytoplasmic staining of XIAP (XIAP-C), was the apoptotic marker which correlated significantly with IDC patients' shortened overall survival. Univariate survival analysis disclosed that patient age, tumor size, lymph node status and XIAP-N had prognostic significance. Nevertheless, it was demonstrated by multivariate survival analysis that only patient age, lymph node status and XIAP-N were independent prognostic factors.

It had been introduced previously that XIAP and Survivin, two important caspase inhibitors of IAP family, were comitantly overexpressing in several kinds of breast cancer cell and oweing to elevated resistance to chemotherapeutics[17,18]. Even more, Survivin was an independent predictor of short-term survival in poor prognostic breast cancer patients[26]. All results stated above called attention to us that overexpression of XIAP and Survivin were significantly correlated with carcinogenesis, progression and prognosis of breast cancer, and the two molecules played similar role in several aspects in breast cancer. Therefore, we concluded XIAP, like Survivin, probably was a new independent prognostic biomarker of breast cancer although it was different that only XIAP nuclear labeling, but not cytoplasm staining, had prognostic significance. This condition was similar to previous reports that Survivin nuclear labeling was a prognostic biomarker of breast cancer and superficial urothelial carcinoma of urinary bladder[29,30].

There was another interesting result in our study that immunoscore of Smac was prevalent in HER2 positive group than negative group, and apoptosis index was positively correlated with HER2 protein expression. Someone believed that apoptosis was decreasing in malignant tumors. But in fact, increasing apoptotic tumor cells could usually be found in malignancies, especially for tumors with high proliferating rate. On the other hand, overexpression of oncogene HER2 contributed to breast carcinogenesis. So, it's not difficult for us to understand that apoptosis index was positively correlated with HER2 protein expression in our study. Our result also consisted with Hinnis' study that AI was not correlated with patient age, tumor size, lymph node status, histologic grading and expression of XIAP, Smac, ER and PR protein[26]. At the same time, our following up data revealed similar result with previous reports that AI was not correlated with IDC patients' prognosis[31,32]. However, why there was prevalent Smac in oncogene HER2 positive group? The relationship between the two parameters had not been reported before, and both mechanism and significance were not clear too. Maybe, this point would be a new target for us to perform a large sample study in the future.

As discussed above, XIAP was a potent protein for apoptosis inhibition and Smac was an important negative regulator of the former. Disturbed balance of expression between XIAP and Smac probably contributed to carcinogenesis and XIAP positive nuclear labeling was a sign of unfavourable prognosis in breast invasive ductal carcinoma. It was important for us to demonstrate the XIAP nuclear staining is genuine, and we were going to practice it in breast cancer cell lines in following study. Further more, Relationship among Smac, HER2 and apoptosis index would be explored in the following study too.

## Conclusion

Disturbed balance of expression between XIAP and Smac probably contributed to carcinogenesis and XIAP-N was a new independent prognostic biomarker of breast invasive ductal carcinoma.

## Competing interests

This paper has never been published and is not under simultaneous review by another journal. All authors have read and approved to submit it to your journal. There is no conflict of interest of any authors in relation to the submission.

## Authors' contributions

YZ, YT and JZ participated in selecting cases, interpretation of results and writing of the manuscript. YZ, FL, BP and CZ carried out the immunohistochemistry, western blot and TUNEL experiment. HZ and RF collected the clinical details of the patients and carried out statistical analysis. All authors read and approved the final manuscript.

## References

[B1] Yan Y Mahotka C Heikaus S Shibata T Wethkamp N Liebmann J Suschek CV Guo Y Gabbert HE Gerharz CD Ramp U Disturbed balance of expression between XIAP and Smac/DIABLO during tumour progression in renal cell carcinomas Br J Cancer 2004 91 7 1349 1357 10.1038/sj.bjc.6602127 15328523 PMC2409908

[B2] Jemal A Siegel R Ward E Hao Y Xu J Thun MJ Cancer statistics, 2009 CA Cancer J Clin 2009 59 4 225 249 10.3322/caac.20006 19474385

[B3] Lee TJ Lee JT Park JW Kwon TK Acquired TRAIL resistance in human breast cancer cells are caused by the sustained cFLIP(L) and XIAP protein levels and ERK activation Biochem Biophys Res Commun 2006 351 4 1024 1030 10.1016/j.bbrc.2006.10.163 17097066

[B4] Peng XH Karna P O'Regan RM Liu X Naithani R Moriarty RM Wood WC Lee HY Yang L Down-regulation of inhibitor of apoptosis proteins by deguelin selectively induces apoptosis in breast cancer cells Mol Pharmacol 2007 71 1 101 111 17035597 10.1124/mol.106.027367

[B5] Zhang Y Wang Y Gao W Zhang R Han X Jia M Guan W Transfer of siRNA against XIAP induces apoptosis and reduces tumor cells growth potential in human breast cancer in vitro and in vivo Breast Cancer Res Treat 2006 96 3 267 277 10.1007/s10549-005-9080-0 16341821

[B6] Lima RT Martins LM Guimaraes JE Sambade C Vasconcelos MH Specific downregulation of bcl-2 and xIAP by RNAi enhances the effects of chemotherapeutic agents in MCF-7 human breast cancer cells Cancer Gene Ther 2004 11 5 309 316 10.1038/sj.cgt.7700706 15031723

[B7] Holcik M Gibson H Robert G XIAP: Apoptotic brake and promising therapeutic target Apoptosis 2001 6 4 253 261 10.1023/A:1011379307472 11445667

[B8] Bilim V Kasahara T Hara N Takahashi K Tomita Y Role of XIAP in the malignant phenotype of transitional cell cancer(TCC) and therapeutic activity of XIAP antisense oligonucleotides against multidrug-resistant TCC in vitro Int J Cancer 2003 103 1 29 37 10.1002/ijc.10776 12455050

[B9] Arnt CR Chiorean MV Heldebrant MP Gores GJ Kaufmann SH Synthetic Smac/DIABLO Peptides Enhance the Effects of Chemotherapeutic Agents by Binding XIAP and clAP1 in Situ J Biol Chem 2002 277 46 44236 44243 10.1074/jbc.M207578200 12218061

[B10] Sun H Nikolovska-Coleska Z Lu J Qiu S Yang CY Gao W Meagher J Stuckey J Wang S Design, synthesis, and evaluation of a potent, cell-permeable, conformationally constrained second mitochondria derived activator of caspase (Smac) mimetic J Med Chem 2006 49 26 7916 7920 10.1021/jm061108d 17181177

[B11] Zobel K Wang L Varfolomeev E Franklin MC Elliott LO Wallweber HJ Okawa DC Flygare JA Vucic D Fairbrother WJ Deshayes K Design, synthesis, and biological activity of a potent Smac mimetic that sensitizes cancer cells to apoptosis by antagonizing IAPs ACS Chem Biol 2006 1 8 525 33 Erratum in: *ACS Chem Biol *2006 1 (9):601. 10.1021/cb600276q 17168540

[B12] Bockbrader KM Tan M Sun Y A small molecule Smac-mimic compound induces apoptosis and sensitizes TRAIL- and etoposide-induced apoptosis in breast cancer cells Oncogene 2005 24 49 7381 7388 10.1038/sj.onc.1208888 16044155

[B13] Nielsen TO Hsu FD O'Connell JX Gilks CB Sorensen PH Linn S West RB Liu CL Botstein D Brown PO van de Rijn M Tissue microarray validation of epidermal growth factor receptor and SALL2 in synovial sarcoma with comparison to tumors of similar histology Am J Pathol 2003 163 1449 1456 10.1016/S0002-9440(10)63502-X 14507652 PMC1868308

[B14] Rajcan-Separovic E Liston P Lefebvre C Korneluk RG Assignment of human inhibitor of apoptosis protein(IAP) genes xiap, hiap-1, and hiap-2 to chromosomes Xq25 and 11q22-q23 by fluoreseence in situ hybridization Genomics 1996 37 3 404 406 10.1006/geno.1996.0579 8938457

[B15] Riedl SJ Renatus M Schwarzenbacher R Zhou Q Sun C Fesik SW Liddington RC Salvesen GS Structural basis for the inhibition of caspase-3 by XIAP Cell 2001 104 5 791 800 10.1016/S0092-8674(01)00274-4 11257232

[B16] Tamm I Kornblau SM Segall H Krajewski S Welsh K Kitada S Scudiero DA Tudor G Qui YH Monks A Andreeff M Reed JC Expression and prognostic significance of IAP family genes in human cancers and myeloid leukemias Clinical Cancer Research 2000 6 5 1796 1803 10815900

[B17] Shi Z Liang YJ Chen ZS Wang XH Ding Y Chen LM Fu LW Overexpression of Survivin and XIAP in MDR cancer cells unrelated to P-glycoprotein Oncol Rep 2007 17 4 969 976 17342344

[B18] Yang L Cao Z Yan H Wood WC Coexistence of high levels of apoptotic signaling and inhibitor of apoptosis proteins in human tumor cells: implication for cancer specific therapy Cancer Res 2003 63 20 6815 6824 14583479

[B19] Nomura T Mimata H Takeuchi Y Yamamoto H Miyamoto E Nomura Y The X-linked inhibitor of apoptosis protein inhibits taxol induced apoptosis in LNCaP cells Urol Research 2003 31 1 37 44 10.1007/s00240-003-0300-y 12624662

[B20] Du CY Li YC Smac: a mitochondrial protein that promotes cytochrome C-independent caspase activation by eliminating IAP inhibition Cell 2000 102 33 42 10.1016/S0092-8674(00)00008-8 10929711

[B21] Hasenjager A Gillissen B Muller A Normand G Hemmati PG Schuler M Dorken B Daniel PT Smac induces cytochrome c release and apoptosis independently from Bax/Bcl-x(L) in a strictly caspase-3-dependent manner in human carcinoma cells Oncogene 2004 23 26 4523 4535 10.1038/sj.onc.1207594 15064710

[B22] Huang Y Rich RL Myszka DG Wu H Requirement of both the second and third BIR domains for the relief of X-linked inhibitor of apoptosis protein (XIAP)-mediated caspase inhibition by Smac J Biol Chem 2003 278 49 49517 49522 10.1074/jbc.M310061200 14512414

[B23] Anne M Verhagen Ekert Identification of DIABLO, a mammalian protein that promotes apoptosis by binding to and antagonizing IAP protein Cell 2000 102 43 53 10.1016/S0092-8674(00)00009-X 10929712

[B24] Srinivasula SM Gupta S Datta P Zhang Z Hegde R Cheong N Fernandes-Alnemri T Alnemri ES Inhibitor of apoptosis proteins are substrates for the mitochondrial sorine protease Omi/HtrA2 J Biolo Chem 2003 278 34 31469 31472 10.1074/jbc.C300240200 12835328

[B25] Yoo NJ Kim HS Kim SY Park WS Park CH Jeon HM Jung ES Lee JY Lee SH Immunohistochemical Analysis of Smac/DIABIO Exprcssion in Human Carcinomas and Sarcomas APMIS 2003 111 3 382 388 10.1034/j.1600-0463.2003.t01-1-1110202.x 12752217

[B26] Hinnis AR Luckett JC Walker RA Survivin is an independent predictor of short-term survival in poor prognostic breast cancer patients Br J Cancer 2007 96 4 639 645 10.1038/sj.bjc.6603616 17285125 PMC2360044

[B27] Ramp U Krieg T Caliskan E Mahotka C Ebert T Willers R Gabbert HE Gerharz CD XIAP expression is an independent prognostic marker in clear cell renal carcinoma Hum Pathol 2004 35 8 1022 1028 10.1016/j.humpath.2004.03.011 15297970

[B28] Jaffer S Orta L Sunkara S Sabo E Burstein DE Immunohistochemical detection of antiapoptotic protein X-linked inhibitor of apoptosis in mammary carcinoma Hum Pathol 2007 38 6 864 870 10.1016/j.humpath.2006.11.016 17350670

[B29] Brennan DJ Rexhepaj E O'Brien SL McSherry E O'Connor DP Fagan A Culhane AC Higgins DG Jirstrom K Millikan RC Landberg G Duffy MJ Hewitt SM Gallagher WM Altered cytoplasmic- to-nuclear ratio of survivin is a prognostic indicator in breast cancer Clin Cancer Res 2008 14 9 2681 2689 10.1158/1078-0432.CCR-07-1760 18451232 PMC7295090

[B30] Yin W Chen N Zhang Y Zeng H Chen X He Y Wang X Zhou Q Survivin nuclear labeling index: a superior biomarker in superficial urothelial carcinoma of human urinary bladder Modern Pathology 2006 19 1487 1497 16892011 10.1038/modpathol.3800675

[B31] Schöndorf T Göhring UJ Becker M Hoopmann M Schmidt T Rützel S Rein DT Ulrich U Fechteler R Bersch A Mallmann P Valter MM High apoptotic index correlates to p21 and p27 expression indicating a favorable outcome of primary breast cancer patients, but lacking prognostic significance in multivariate analysis Pathobiology 2004 71 4 217 222 10.1159/000078676 15263811

[B32] Sirvent JJ Aguilar MC Olona M Pelegrí A Blázquez S Gutiérrez C Prognostic value of apoptosis in breast cancer (pT1-pT2). A TUNEL, p53, bcl-2, bag-1 and Bax immunohistochemical study Histol Histopathol 2004 19 3 759 770 15168338 10.14670/HH-19.759

